# New insights into the (epi)genetics of twinning

**DOI:** 10.1093/humrep/dead131

**Published:** 2023-12-05

**Authors:** Jenny van Dongen, Nikki Hubers, Dorret I Boomsma

**Affiliations:** Netherlands Twin Register, Department of Biological Psychology, Amsterdam Reproduction and Development Research Institute, Vrije Universiteit Amsterdam, Amsterdam, The Netherlands; Netherlands Twin Register, Department of Biological Psychology, Amsterdam Reproduction and Development Research Institute, Vrije Universiteit Amsterdam, Amsterdam, The Netherlands; Netherlands Twin Register, Department of Biological Psychology, Amsterdam Reproduction and Development Research Institute, Vrije Universiteit Amsterdam, Amsterdam, The Netherlands

**Keywords:** chorionicity, early development, epigenome-wide association study, genome-wide association study, vanishing twin, IVF, ART, Twinning Genetics Consortium, zygosity, multiple pregnancy

## Abstract

Spontaneous dizygotic (DZ) twins, i.e. twins conceived without the use of ARTs, run in families and their prevalence varies widely around the globe. In contrast, monozygotic (MZ) twins occur at a constant rate across time and geographical regions and, with some rare exceptions, do not cluster in families. The leading hypothesis for MZ twins, which arise when a zygote splits during preimplantation stages of development, is random occurrence. We have found the first series of genes underlying the liability of being the mother of DZ twins and have shown that being an MZ twin is strongly associated with a stable DNA methylation signature in child and adult somatic tissues. Because identical twins keep this molecular signature across the lifespan, this discovery opens up completely new possibilities for the retrospective diagnosis of whether a person is an MZ twin whose co-twin may have vanished in the early stages of pregnancy. Here, we summarize the gene finding results for mothers of DZ twins based on genetic association studies followed by meta-analysis, and further present the striking epigenetic results for MZ twins.

## Graphical Abstract

**Graphical Abstract:**
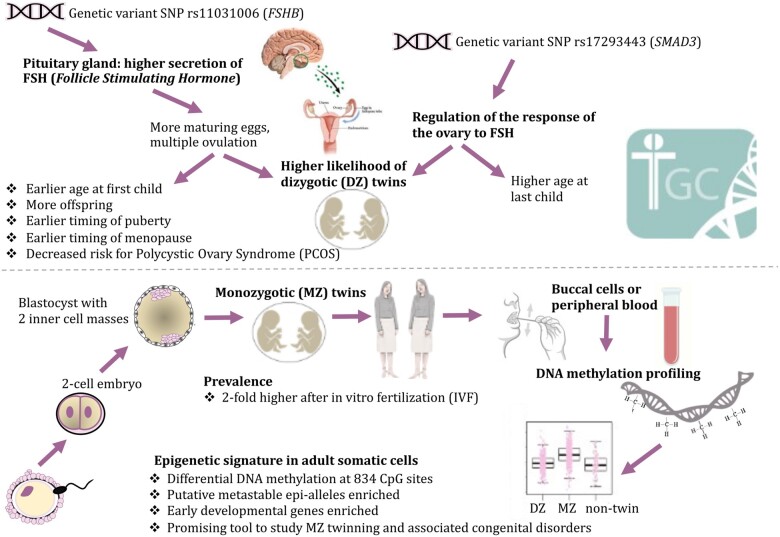
**Recent findings regarding the genetic susceptibility to being the mother of dizygotic (DZ) twins (top) and an epigenetic signature associated with being a monozygotic (MZ) twin (bottom).** TGC (logo): Twinning Genetics Consortium; SNP: single-nucleotide polymorphism.

## Introduction

Globally, twin birth rates (defined as the proportion of twin deliveries per 1000 deliveries) varied from less than 10 twin deliveries per 1000 deliveries in poorer regions of Latin America and South-East Asia to up to 30 deliveries in several African countries in the period of 2010–2015 (Monden *et al.*, 2021). Most of the variation across countries and also over time is due to variation in dizygotic (DZ) twinning (Bortolus *et al.*, 1999; Monden *et al.*, 2021). In this article, we use the term ‘twinning’ to refer to the process that gives rise to two or more offspring in a single pregnancy.

DZ twins arise after double ovulation when both oocytes are fertilized and become embryos. Spontaneous DZ twinning is familial, is associated with maternal age and parity, and shows striking global differences (Hall, 2003; Hoekstra *et al.*, 2008; Smits and Monden, 2011). The frequency of DZ twins has risen over time due to the use of ART and increasing maternal age (Monden *et al.*, 2021). By contrast, monozygotic (MZ) twins rarely run in families, and their prevalence is fairly similar across the world and over time (3–4 MZ pairs per 1000 births) (Bulmer, 1970; Bortolus *et al.*, 1999), and stable with respect to the mother’s age (Bulmer, 1970; Hamamy *et al.*, 2004; Hoekstra *et al.*, 2008). A prevailing hypothesis therefore is that MZ twinning occurs at random. MZ twins arise when the progeny of a single fertilized oocyte divides early in development to give rise to two or more embryos. The underlying mechanism of MZ twinning has been an enigma of human developmental biology. The frequency of MZ twinning is slightly increased following the use of ART (Vitthala *et al.*, 2009). In particular, prolonged embryo culture (i.e. blastocyst culture) in IVF is associated with an approximately 2-fold increase in the frequency of MZ twinning (Vitthala *et al.*, 2009; Ding *et al.*, 2018; Hviid *et al.*, 2018; Busnelli *et al.*, 2019; Hattori *et al.*, 2019), but the mechanism responsible for this effect is unknown.

Multiple pregnancies are associated with an increased risk of morbidity and mortality of mother and offspring (D’Alton and Breslin, 2020). Understanding the mechanisms involved can be valuable in managing the preventable risks associated with twinning. Here, we review recent insights from genetic and epigenetic studies of twinning. We first summarize recent insights from genetic studies of DZ twinning, in which the liability for DZ twin births is considered a feature of the mother. Secondly, we discuss recent findings from epigenetic studies showing that MZ twins carry a distinct DNA methylation signature. This signature might lead to novel insights into the process of MZ twinning.

## Novel insights from genetic studies of DZ twinning

A genetic component to DZ twinning (Bulmer, 1970) has been repeatedly observed in multi-generation data, which showed that DZ twin births cluster in families (Lewis *et al.*, 1996; Meulemans *et al.*, 1996). This genetic component likely includes maternal genes that affect a double ovulation as well as maternal genes influencing the ability to successfully carry a multiple pregnancy. Initially there was optimism that strong animal models for DZ twinning, for example the work done in sheep, would lead to the identification of the genes in humans by candidate gene studies. It was thought that the well-characterized loci leading to multiple ovulation, in e.g. Booroola merino ewes, would hold the key to unravelling multiple ovulation in humans (Wilson *et al.*, 2001). Mutations in three genes, *GDF9*, *BMP15*, and *BMPRB1* in the TGFβ pathway are important for the growth of ovarian follicles and affect twinning rates in sheep. In humans, rare and low-frequency variants in these genes are unlikely to account for more than a small fraction of the risk in DZ twinning. Palmer *et al.* (2006) and Luong *et al.* (2011) looked at mutations in these genes in mothers of DZ twins (MoDZT) from twin dense families. Rare variants in the coding regions were only seen in *GDF9* and these were of unknown significance (Palmer *et al.*, 2006). Based on these results, the researchers concluded that variation in the TGFβ pathway will have only a limited contribution on DZ twinning rates in humans.

Early candidate gene studies in humans suggested the highly polymorphic protease inhibitor (PI) locus (on chromosome 14), coding for alpha-1-antitrypsin (AAT), as a strong candidate. Some of the Z- and S-alleles of this locus associated with DZ twinning (Lieberman *et al.*, 1979; Clark and Martin, 1982; Boomsma *et al.*, 1992). However these findings still need confirmation. Gajbhiye *et al.* (2018) reviewed most of the candidate gene studies and concluded that we ‘are still far away from resolving the genetics on DZ twinning’.

The search for common genetic variants, i.e. genes with a minor allele frequency of 1–50%, that underlie DZ twinning became feasible with genome-wide association studies (GWAS). Here, genetic variation is measured by large numbers of single-nucleotide polymorphisms (SNPs) across the entire genome. In 2016, Mbarek *et al.* (2016) analysed SNP data from 1980 mothers of spontaneous DZ twins (i.e. DZ twins born without the use of reproductive technologies) and 12 953 controls. The study identified an association with DZ twinning for two SNPs that replicated in the deCODE Icelandic database. One finding was for follicle-stimulating hormone beta subunit (*FSHB*) and the other one was for SMAD family member 3 (*SMAD3*). The risk alleles close to *FSHB* and in *SMAD3* increased the frequency of twin births in the Icelandic population by 18% and 9%, respectively (Mbarek *et al.*, 2016). Genes encoding FSH had always been strong candidates for DZ twinning.

*SMAD3* (chromosome 15) was an entirely novel finding for DZ twinning. The lead SNP is located in the first intron of *SMAD3*. *SMAD3* is highly expressed in human ovaries, and stimulates proliferation of granulosa cells and steroidogenesis. The chromosome 15q22.33 region also harbours SMAD family member 6 (*SMAD6*). *SMAD3* and *SMAD6* are both part of the transforming growth factor-β (TGFβ)/BMP signalling system. The human 15q22.33 region harbouring *SMAD3* and *SMAD6* is homologous to a region on chromosome 10 in the bovine genome. This region is associated with twinning and increased ovulation rates in cattle (Kirkpatrick and Morris, 2015; Kamalludin *et al.*, 2018). The SNPs that are associated with DZ twinning also influence other reproductive traits in women, including earlier age at first child, higher total lifetime number of children, earlier age at natural menopause, and lower risk of polycystic ovary syndrome (PCOS) (Hayes *et al.*, 2015; Lyle *et al.*, 2023). The rs17293443-C allele in *SMAD3* was associated only with a later age at last child. The roles of rs11031006, which is located in a highly conserved region upstream of the human *FSHB* gene, for multiple aspects of fertility has been discussed (Bohaczuk *et al.*, 2021). These authors hypothesized that the region regulates *FSHB* transcription and found that the rs11031006 minor allele upregulated *FSHB* transcription via increased steroidogenic factor 1 (SF1) binding to the enhancer, indicating that rs11031006 can modulate FSH levels. A larger GWAS meta-analysis (GWAMA), now including 8265 MoDZT and 26 252 DZ twins themselves (‘proxy’ for the trait ‘being the mother of DZ twins’) as well as 682 000 controls replicated the association with *FSHB* and *SMAD3* (Mbarek *et al.*, 2023). The meta-analysis also identified meaningful genetic regions involved in female fertility such as gonadotropin-releasing hormone 1 (*GNRH1*) and the follicle-stimulating hormone receptor (*FSHR). GNRH1* and *FSHR* are involved in the same hormonal pathway as *FSHB* eventually leading to ovulation (Casarini and Crépieux, 2019). Other genomics regions with a so far unknown associations with female fertility, such as *ZFPM1* and *IPO8*, were also identified. Gene-based tests highlighted *ARL14EP* as the causal gene in the region of *FSHB* on chromosome 11. *ARL14EP* was previously associated with another reproductive phenotype, PCOS (Lyle *et al.*, 2023), which emphasizes the relationship between DZ twinning and other female fertility traits. However, the mechanism(s) by which *ARL14EP* influences fertility is still unclear. Even though the gene-based tests highlight *ARL14EP* as the causal gene, they do not rule out *FSHB* and both genes are likely to influence the processes leading to DZ twinning. Several hits were just below the genome-wide significance threshold and more genetic regions are expected to be involved in DZ twinning. An increase in sample size and the addition of more ancestries to the so far only European study cohorts will help to uncover other genomic regions, especially since the prevalence of DZ twins varies highly across ancestries (Monden *et al.*, 2021).

## MZ twinning, ART and epigenetics

There is very limited evidence for a genetic contribution to MZ twinning. The frequency of MZ twins has been reported to be 2.25 times higher after ART compared to spontaneous conceptions (Vitthala *et al.*, 2009), with prolonged embryo culture (i.e. blastocyst culture) in IVF being the greatest risk factor (Vitthala *et al.*, 2009; Ding *et al.*, 2018; Hviid *et al.*, 2018; Busnelli *et al.*, 2019; Hattori *et al.*, 2019). The frequency of MZ twins is 10-fold higher among patients with Beckwith–Wiedemann Syndrome (Weksberg, 2002; Bliek *et al.*, 2009), an imprinting disorder, for which the risk also is increased after ART (Doornbos *et al.*, 2007; Brioude *et al.*, 2018). ART can affect epigenetic marks in the foetus and placenta, with both human and animal studies reporting changes in DNA methylation at imprinted genes, repetitive elements and other genome-wide loci (Mani *et al.*, 2020). Proper epigenetic programming is potentially impacted by many aspects of ART, including embryo manipulation, culture and transfer, and the exposure of gametes and/or embryos to alterations in hormone levels, temperature, pH, and oxygen tension (Mani *et al.*, 2020).

MZ twins arise around critical periods for epigenetic (re)programming. A first wave of global epigenetic reprogramming occurs during gametogenesis (Hales *et al.*, 2011). Subsequently, shortly after fertilization the next round of epigenetic reprogramming starts and the pre-implantation embryo undergoes multiple waves of global DNA demethylation, followed by *de novo* methylation (Zhu *et al.*, 2018). These waves coincide with initially pluripotent cells differentiating into distinct cell lineages. The correct establishment of DNA methylation patterns is vital for embryonic development (Jones, 2012). Epigenetic regulation of gene expression controls the functional activity of gene promoters, enhancers, and other regulatory regions of the genome. In DNA methylation, a major mechanism of epigenetic regulation, a methyl group (CH_3_) is added on cytosine–phosphate–guanine (CpG) dinucleotides in the genome. (Adenine and thymine, cytosine and guanine are the four building blocks of the DNA.) The transfer of a methyl group to the C5 position of the cytosine at specific genomic positions can regulate gene expression and is crucial for tissue-specific processes. DNA methylation and other repressive epigenetic modifications, for example histone modifications, are responsible for suppressing embryonic genes in differentiated cells and maintaining parental-specific expression of imprinted genes (Jones, 2012; Slieker *et al.*, 2015).

Inspired by previously reported associations between MZ twinning, artificial reproductive technologies (ART), and imprinting disorders, we turned to a genome-wide epigenetic association study (EWAS) of being an MZ twin. Via meta-analysis, epigenetic data from four countries were combined (van Dongen *et al.*, 2021a).

## Novel insights from the large epigenetic association meta-analysis of MZ twinning

DNA methylation data from blood and buccal samples were analysed in six independent twin cohorts: two from the Netherlands, two from UK, a cohort from Finland, and one from Australia (van Dongen *et al.*, 2021a). DZ twins who, like MZ twins (but unlike singletons), experience similar prenatal conditions were the control group, thus controlling for possible effects of sharing a womb with a co-twin. The study did not differentiate between MZ twins born after a spontaneous pregnancy versus twins conceived with the use of ART, since this information was not available for all twins in this study. However the majority of twins were born before the introduction of ARTs.

DNA methylation profiles were obtained with Illumina methylation arrays, mainly the Illumina Infinium 450k array, which measures ∼450 000 CpG sites and with the newer Illumina Infinium EPIC array which contains ∼850 000 CpG sites. The utilization of DNA methylation microarrays allows investigation of site-specific DNA methylation across an individual’s genome while also allowing for high sample throughput. Pipelines for quality control (QC), and pre-processing and analysis of DNA methylation array data were developed in the Netherlands by the Dutch Biobank-Based Integrative Omics Study (BIOS) consortium (Sinke *et al.*, 2022).

The first step was a discovery analysis in DNA methylation data (from whole blood samples) from adult MZ and DZ twins (mean age 35 years) from the Netherlands Twin Register (NTR). The analysis included one randomly selected twin from each pair of 924 MZ twin pairs (924 individuals in total) and 1033 DZ twin individuals (including 419 twin pairs where both twins of a pair were included). This analysis identified 243 differentially methylated positions (DMPs) between MZ twins and DZ twins. Effect sizes ranged from a 0.3% to 6% difference in DNA methylation levels. Replication analyses were performed in four independent cohorts with DNA isolated from adult blood samples, with mean ages ranging from 18 to 58 years, and revealed strong concordance of effects: the correlations (*r*) of effect sizes ranged from 0.84 to 0.97. The effects identified in DNA samples from the adult twins also showed strong concordance of effects in children's DNA samples derived from another tissue of a distinct embryonic cell lineage, buccal cells (*r *=* *0.87). Buccal samples consist mainly of epithelial cells (Theda *et al.*, 2018; van Dongen *et al.*, 2018), which originate from the ectodermal lineage, while white blood cells originate from the mesodermal cell lineage. Several sensitivity analyses were performed: DNA methylation in MZ twins was compared to DNA methylation in non-twins (parents and siblings); DNA methylation in DZ twins was compared to DNA methylation in non-twins; the analysis was repeated in complete twin pairs and in a randomly selected twin from each pair; and the analysis was repeated separately for male and female twin pairs. The difference in DNA methylation between MZ twins and other groups was highly consistent across all comparisons, while DZ twins did not differ markedly from non-twins, indicating that the results from the primary EWAS mainly reflect differential DNA methylation in MZ twins.

**Figure 1.**
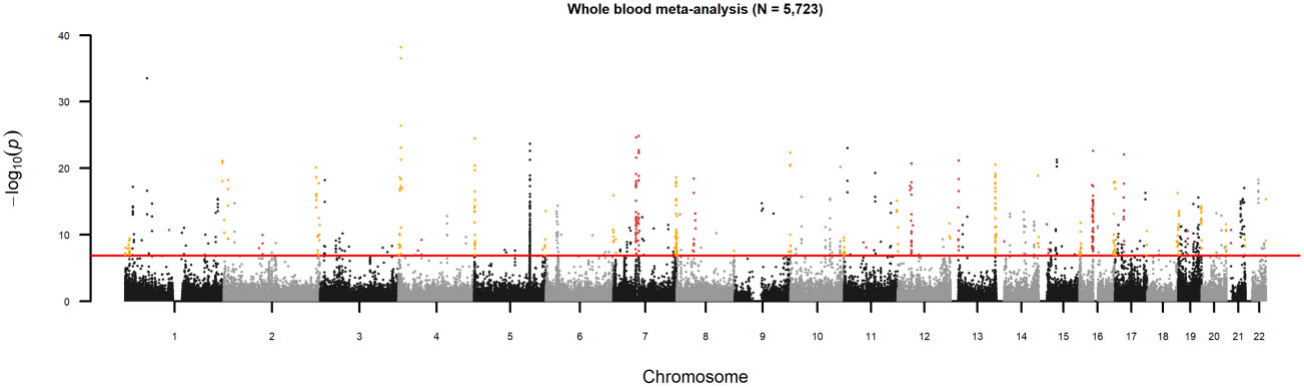
**Monozygotic (MZ) twinning DMPs identified in a meta-analysis of data from 5723 twins.** Reproduced with permission, from Nature Communications 2021 (van Dongen *et al.*, 2021a). Manhattan plot of the EWAS meta-analysis based on whole blood DNA methylation data from five twin cohorts (total sample size = 5723) that identified 834 MZ-DMPs. The red horizontal line denotes the epigenome-wide significance threshold (Bonferroni correction). Dark red dots highlight significant DMPs near centromeres. Orange dots highlight significant DMPs near telomeres. DMPs: differentially methylated positions; EWAS: epigenome-wide association study.

All blood EWAS results from all five of the independent twin cohorts were subsequently combined in a meta-analysis (total sample size = 5723); this revealed 834 CpGs (Fig. 1) that were significantly different between MZ twins and DZ twins. We refer to these as ‘MZ-DMPs’: of which 497 (60%) had a lower methylation level in MZ twins (MZ-hypo-DMPs) while 337 (40%) had a higher methylation level (MZ-hyper-DMPs).

## MZ-DMPs are more stable across time and tissues compared to other methylation sites

To gain some insight into the properties of MZ-DMPs, we evaluated their correlation in longitudinal blood samples, their correlation across different tissues, and their heritability (i.e. the extent to which the variation in DMPs is explained by inherited DNA variation).

A characterization of the correlation between longitudinal DNA methylation levels in repeat blood samples, collected at an average interval of 5 years, showed that MZ-DMPs had on average an intermediate methylation level in blood (mean = 0.52) and were highly stable over time (mean longitudinal correlation = 0.85). A high stability is expected for sites whose methylation level is established early in life and this indicates a high reliability of the DNA methylation measurement.

To examine the correlation across tissues, we looked at the correlation between blood and buccal samples from the same individuals (van Dongen *et al.*, 2016), and between blood and post-mortem brain samples, based on previously published methylation data from matched blood and brain samples (Hannon *et al.*, 2015). Some of the CpGs showed strong correlations between blood and buccal cell methylation levels, with the mean correlation across all CpGs estimated to be 0.44, and between blood and brain (mean r = 0.27–0.43, for four different brain regions). A recent study characterized CpGs that are hypervariable across multiple tissues (hvCpGs) but whose methylation level tends to correlate across tissues derived from different germ layers (Derakhshan *et al.*, 2022). At these CpGs, the methylation level is likely to be established in the early embryo (i.e. prior to the formation of the different germ layers) (Derakhshan *et al.*, 2022). Interestingly, a large proportion (37%) of the MZ-DMPs was among the list of hvCpGs identified in this recent paper (Derakhshan *et al.*, 2022). Similarly, strong enrichment was observed for putative human metastable epi-alleles (MEs) among the MZ-DMPs. MEs are loci with highly variable methylation states (even across genetically identical individuals), but within the individual, the methylation state at such loci is systemic (stable across tissues) (Rakyan *et al.*, 2002). MEs are enriched near certain classes of transposable elements, including long interspersed nuclear elements (LINEs) and endogenous retroviruses (ERVs) (Silver *et al.*, 2015; Kessler *et al.*, 2018). The methylation level at MEs is thought to be influenced by genotype, periconceptional environmental exposures, and presumably stochastic processes. MEs were previously described to exhibit ‘epigenetic super-similarity’ between MZ co-twins (van Baak *et al.*, 2018). The enrichment of MEs among the MZ-DMPs is striking, because their methylation state is established around the time of implantation of the embryo (Kessler *et al.*, 2018). This coincides with the time when the majority of MZ twinning events are thought to occur (giving rise to monochorionic diamniotic MZ twins).

Finally, we looked at heritability, i.e. whether the DNA methylation patterns we observed might be influenced by genomic variation (DNA sequence variation). To this end, we compared the degree of resemblance of MZ and DZ twins (van Dongen *et al.*, 2016). The pattern of twin correlations in blood showed correlations of the methylation level of the 834 MZ-DMP sites in MZ twins being almost three times larger on average compared to that in DZ twins (mean MZ correlation = 0.58, mean DZ correlation = 0.20), implying a significant heritability, which is on average much larger than the mean heritability of 19% of all genome-wide autosomal methylation sites (van Dongen *et al.*, 2016). This pattern of twin correlations is consistent with non-additive genetic effects (dominance or epistasis), or could arise if the methylation state is established before the embryo splits in the case of MZ twins, who therefore have similar methylation levels after splitting, while it is established separately in the two embryos in the case of DZ twins, who therefore have less similar DNA methylation levels (van Baak *et al.*, 2018).

## Chorionicity

For 184 MZ twins pairs in the Netherlands Twin Register part of the EWAS project, data on chorionicity were available via linking the NTR child cohort data to the Pathological Anatomy National Automatic Archive of the Netherlands (PALGA) (van Beijsterveldt *et al.*, 2016). It has been hypothesized, though never empirically demonstrated, that if the zygote splits shortly after fertilization, dichorionic MZ twins arise, whereas splitting ≥3 days after fertilization results in monochorionic twins (Beck *et al.*, 2021). Buccal DNA methylation was compared across three groups: (i) dichorionic MZ twins (∼1/3 of all MZ twins, and presumably arising from an early splitting event), (ii) monochorionic diamniotic MZ twins (∼2/3 of all MZ twins), and (iii) monochorionic monoamniotic MZ twins (<1% of MZ twins, and believed to originate from a late splitting event) (Machin, 2005). Overall, the correlations between MZ co-twins from these three groups did not show differences when looking at genome-wide methylation sites. In contrast, the MZ-DMPs showed a pattern of stronger resemblance in MZ monochorionic monoamniotic twins (mean *r *=* *0.57), and somewhat smaller resemblances in monochorionic diamniotic twins (mean *r *=* *0.45), and MZ dichorionic twins (mean *r *=* *0.41). The small mean differences were driven by a subset of MZ-DMPs that showed larger differences. One explanation for this pattern could be that some of the methylation state is established prior to the splitting of monochorionic twins. At loci where the methylation state is established very early, later splitting twins would be more likely to inherit the same methylation state through mitosis than the twins who split earlier, prior to establishment of the methylation state. However, the groups were small and the finding requires replication.

## Telomeric and centromeric regions, early embryonic development, and cell adhesion

Looking at the genomic locations of the MZ DMPs (Fig. 1), the enrichment near telomeres and centromeres is striking: DMPs with a lower methylation level in MZ twins (MZ-hypo-DMPs) occurred much more often than by chance near telomeres (45% of all MZ-hypo-DMPs) and DMPs with a higher methylation level in MZ twins (MZ-hyper-DMPs) clustered near centromeres (41% of MZ-hyper-DMPs). MZ-hypo-DMPs and MZ-hyper-DMPs were also enriched in intergenic regions and CpG islands. Strong enrichment for MZ-hypo-DMPs was further seen in Polycomb-repressed regions (Kundaje *et al.*, 2015), typically associated with transcriptionally silenced developmental genes, and characterized by H3K27me3 (Histone H3 lysine 27 tri-methylation). MZ-hyper DMPs, on the other hand, were enriched in two other chromatin states associated with condensed DNA containing a broader group of transcriptionally repressed regions, namely heterochromatin, and the chromatin state associated with ‘ZNF genes and repeats’. Heterochromatin is the tightly packed form of condensed DNA, typically found around the telomeres and centromeres.

Transcription factors (TF) are proteins that initiate and regulate the transcription of their target genes by binding to their specific recognition sequence (i.e. motif) in the DNA. Methylation of TF motifs is a common mechanism associated with target gene expression silencing. To gain some insight into functional consequences of MZ-DMPs, we examined their overlap with TF binding sites in the DNA. MZ-DMPs were enriched within the bindings sites of various TFs expressed in early embryos, including homeobox TFs, which are well-known regulators of gene expression and cell differentiation during early embryonic development. Next, the target genes regulated by the enriched TFs were analysed. The target genes were involved in early embryonic processes including ‘anterior/posterior pattern specification’, ‘chordate embryonic development’, and ‘tube development’. Pathway analyses based on the nearest genes of MZ-DMPs showed enrichment of pathways including ‘cell fate specification’, ‘cell adhesion’, and ‘Wnt signalling’. Future functional studies should examine how these pathways might be connected to MZ twinning.

The published results from the Genetics of DNA Methylation Consortium (Min *et al.*, 2021) contain methylation Quantitative Trait Loci (mQTL); these are loci harbouring genetic variants associated with methylation levels of nearby CpGs (i.e. cis mQTLs) or with methylation levels of distant CpGs (i.e. trans mQTLs). The methylation level at MZ-DMPs was associated with cis and trans mQTLs. Interestingly, trans mQTLs included several key epigenetic modifier loci such as *TRIM28* and *DNMT3B*, and key pluripotency regulators *DPPA4* (Maldonado-Saldivia *et al.*, 2007; Masaki *et al.*, 2007) and *DPPA2* (Hernandez *et al.*, 2018). Trans mQTLs are loci where the genotype is associated with methylation level of a CpG further than 1 Mb from away and these may regulate multiple genes. Looking at the trans mQTLs associated with methylation at MZ-DMPs, the number of MZ-DMPs affected by each trans QTL ranged from 1 to 15.

## Retrospective diagnosis of MZ twinning and congenital disorders

For a number of congenital disorders, a higher rate of MZ twins among affected individuals has been observed, for example, the almost 10-fold higher frequency of MZ twins (Weksberg, 2002; Bliek *et al.*, 2009) in Beckwith–Wiedemann Syndrome (MIM130650) (Myrianthopoulos, 1976; Schinzel *et al.*, 1979; Hall, 2003; Glinianaia *et al.*, 2008; Pharoah and Dundar, 2009; Holmes, 2012) and Amyoplasia (Hall, 2021) and 3–10 times higher frequency of MZ twins amongst infants with different types of neural tube defects (Källén *et al.*, 1994; van Dongen *et al.*, 2021b). Another striking example is Sirenomelia (mermaid syndrome), a rare congenital malformation in which the legs of a baby are fused together. It has been reported that the incidence of this malformation is 100-fold increased in MZ twin births (Davies *et al.*, 1971). For such disorders, it has been hypothesized that affected singletons began life as a pair of MZ twins in the womb, without the mother’s knowledge, the so-called vanishing twin syndrome (Jauniaux *et al.*, 1988; Landy and Keith, 1998). At present, tools to investigate this hypothesis are lacking.

It has been estimated that 12% of pregnancies may start with multiples (either MZ or DZ) in humans, but a large proportion involves loss of all embryos or one vanishing twin, resulting in <2% of multiples being carrying to term (Boklage, 1990). These estimates were derived by curve fitting to recorded survival rates during different embryonic/foetal stages of ultrasonically detected twins as well as recorded frequencies of twins among miscarriages and stillborn births, and subsequent extrapolation to survival probability before clinical pregnancy recognition (Boklage, 1990). Separate estimates for MZ and DZ twins are lacking.

To test how well MZ twin status could be predicted, a DNA methylation-based classifier of MZ twinning was developed. With penalized regression models, our trait of interest, in this case ‘being an MZ twin’ with two levels (yes/no), was regressed on DNA methylation levels at a large number of genomic sites. The aim was to identify the best-predicting subset of DNA methylation sites and obtain a formula to predict being an MZ twin based on DNA methylation levels at these sites. We used elastic regression models that allow for some degree of correlation among DNA methylation sites in the model. Models were trained using 10-fold cross-validation. The predictor achieved an area under the curve (AUC) of 0.77 in the NTR cohort (N∼1000 blood samples in the test dataset) and an AUC of 0.80 in an independent twin dataset from Australia (N = 606 blood samples). The performance was similar for buccal DNA (N = 1237) and for monochorionic or dichorionic MZ twins.

An AUC is a function of sensitivity and specificity. In the test dataset from the Netherlands Twin Register, the sensitivity, the proportion of correctly predicted MZ twins, was up to 84% and the specificity, the proportion of DZ twins correctly classified as non-MZ, was 57%. Up to 63% of family members were correctly classified as non-MZ. In data from Australia, the sensitivity was higher, but the specificity was lower: up to 92% of MZ twins were correctly predicted as MZ twins, up to 47% of DZ twins were correctly predicted as non-MZ, and up to 45% of family members were correctly predicted as non-MZ twins. The predictor was only tested in twin cohorts and the DZ twins and family members in the training and test datasets may have included an unknown number of MZ multiples, if DZ twins or other family members had a vanishing co-twin.

## Future directions in MZ twin research

The MZ DNA methylation score’s predictive accuracy is not 100%, but it may already provide a valuable biomarker for MZ twinning that could be employed to examine the vanishing twin hypothesis of diseases that are strongly linked to MZ twinning, such as Beckwith–Wiedemann Syndrome (Weksberg, 2002; Bliek *et al.*, 2009), Amyoplasia (Hall, 2021), and neural tube defects including anencephaly and spina bifida (van Dongen *et al.*, 2021b) by obtaining the methylation score on patients who present as singletons.

The findings provide compelling hints towards an epigenomic event in MZ twins, but remain observational and can represent a cause, an effect, or a byproduct of the MZ twinning event. The signature was identified in adult twins and replicated in children. Based on these data alone, it is not possible to tell when the signature is established. Further functional studies in cellular or animal models are required to delineate exactly how this signature is connected to MZ twins.

The frequency of MZ twins is increased after ART, with prolonged embryo culture (i.e. blastocyst culture) in IVF being the greatest risk factor (Vitthala *et al.*, 2009; Ding *et al.*, 2018; Hviid *et al.*, 2018; Busnelli *et al.*, 2019; Hattori *et al.*, 2019). The mechanisms underlying this association remain to be unravelled. Studies examining the effect of culturing techniques on the MZ twining epigenetic signature are warranted.

Despite being a valuable tool for research, we believe that the MZ twin epigenetic signature is not quite ready for individual-level prediction. Although we recognize that there is demand for such a test by individuals seeking conclusive evidence that they are a sole-born twin, the specificity of the test is too low for individual-level prediction.

## Conclusion

The discovery of a strong and stable epigenetic signature in MZ twins points to DNA methylation as a biological marker on which functional studies could further focus to elucidate the biology of MZ twinning. The findings for DZ twinning originate from genome-wide association analyses, and an association between a trait and a DNA variant also requires further work to establish the causal variants. It is important to note that the studies were thus far conducted in European twin populations. Future studies in diverse populations will likely contribute to fine mapping of causal variants, and lead to a better understanding of why twinning rates vary so much across countries (Smits and Monden, 2011; Monden *et al.*, 2021). The recent findings already offer multiple possibilities to define at the individual level either a polygenic risk score for DZ twinning in women or a methylation signature of MZ twinning. Intriguing questions remain. For example, is there a phenotypic expression in men of a high a polygenic risk score for DZ twinning? And does the DNA methylation signature of MZ twins have phenotypic consequences?

## Data Availability

There are no new data associated with this article.
